# Extensive deamidation at asparagine residue 279 accounts for weak immunoreactivity of tau with RD4 antibody in Alzheimer’s disease brain

**DOI:** 10.1186/2051-5960-1-54

**Published:** 2013-08-21

**Authors:** Ayaho Dan, Muneaki Takahashi, Masami Masuda-Suzukake, Fuyuki Kametani, Takashi Nonaka, Hiromi Kondo, Haruhiko Akiyama, Takao Arai, David MA Mann, Yuko Saito, Hiroyuki Hatsuta, Shigeo Murayama, Masato Hasegawa

**Affiliations:** 1 Department of Neuropathology and Cell Biology, Tokyo Metropolitan Institute of Medical Science, Setagaya-ku, Tokyo 156-8506, Japan; 2 Histology center, Tokyo Metropolitan Institute of Medical Science, Setagaya-ku, Tokyo 156-8506, Japan; 3 Dementia Research Project, Tokyo Metropolitan Institute of Medical Science, Setagaya-ku, Tokyo 156-8506, Japan; 4 Department of Applied Biological Science, Faculty of Science and Technology, Tokyo University of Science, 2641 Yamazaki, Noda-shi, Chiba-ken 278-8510, Japan; 5 Centre for Clinical and Cognitive Neuroscience, Institute of Brain Behavior and Mental Health, University of Manchester, Salford M6 8HD, UK; 6 Department of Laboratory Medicine, National Center Hospital, NCNP, 4-1-1 Ogawahigashi, Kodaira, Tokyo 187-8502, Japan; 7 Department of Neuropathology, Tokyo Metropolitan Institute of Gerontology, Itabashi-ku, Tokyo 173-0015, Japan

**Keywords:** Alzheimer’s disease, Tau, Deamidation, Aging, Microtubule

## Abstract

**Background:**

Intracytoplasmic inclusions composed of filamentous tau proteins are defining characteristics of neurodegenerative tauopathies, but it remains unclear why different tau isoforms accumulate in different diseases and how they induce abnormal filamentous structures and pathologies. Two tau isoform-specific antibodies, RD3 and RD4, are widely used for immunohistochemical and biochemical studies of tau species in diseased brains.

**Results:**

Here, we show that extensive irreversible post-translational deamidation takes place at asparagine residue 279 **(**N279) in the RD4 epitope of tau in Alzheimer’s disease (AD), but not corticobasal degeneration (CBD) or progressive supranuclear palsy (PSP), and this modification abrogates the immunoreactivity to RD4. An antiserum raised against deamidated RD4 peptide specifically recognized 4R tau isoforms, regardless of deamidation, and strongly stained tau in AD brain. We also found that mutant tau with N279D substitution showed reduced ability to bind to microtubules and to promote microtubule assembly.

**Conclusion:**

The biochemical and structural differences of tau in AD from that in 4R tauopathies found in this study may therefore have implications for prion-like propagation of tau.

## Background

Intracellular inclusions composed of filamentous tau proteins are defining characteristics of many neurodegenerative diseases, including Alzheimer’s disease (AD), Pick’s disease, corticobasal degeneration (CBD), and progressive supranuclear palsy (PSP). Tau is a microtubule-associated protein that stabilizes microtubules and promotes their assembly. In adult human brain, 6 tau isoforms are expressed as a result of mRNA splicing. They are divided into two groups, 3-repeat (3R) and 4-repeat (4R) tau isoforms, according to whether or not exon 10 is expressed. Tau pathologies show clear morphological differences among different diseases or disease types, and different tau isoforms are accumulated in the diseased brains, namely, 6 tau isoforms in AD, 3R tau isoforms in Pick’s disease, and 4R tau isoforms in PSP and CBD [1,2]. In addition, tau in PSP and tau in CBD are biochemically distinguished by the banding pattern of the C-terminal fragments [3]. However, it remains unclear why different tau isoforms accumulate in different diseases and how they lead to the formation of abnormal filamentous structures and pathologies.

Isoform-specific tau antibodies are useful tools for immunohistochemical and biochemical studies of tau species in diseased brains. In particular, RD3 and RD4 [4], which are specific antibodies to 3R and 4R tau isoforms, respectively, have been widely used to investigate tau pathologies [5-7]. One of the present authors (M.H.) had found that the asparagine residue at position 279 (N279), located in the RD4 epitope, was detected mostly as aspartic acid owing to deamidation of asparagine when PHF-tau in AD brains was subjected to protein sequencing and LC/MS/MS analysis after digestion with lysyl endopeptidase [8]. Here, we show that the irreversible post-translational deamidation takes place at N279 (N279D) in the RD4 epitope of tau in AD, but not CBD or PSP, and this modification abrogates the immunoreactivity to RD4. We raised an antiserum against RD4 peptide with N279D in rabbit, and showed that it specifically recognizes 4R tau isoforms regardless of deamidation and strongly stained tau in AD brain. We further show that mutant tau with N279D substitution has a reduced ability to bind to microtubules and to promote their assembly. These results have important implications for immunohistochemical and other studies aimed at understanding the molecular mechanisms of tau accumulation in AD and other tauopathies.

## Results

### Low immunoreactivities of tau in AD and tau deamidated at N279 to RD4

**Figure 1:**
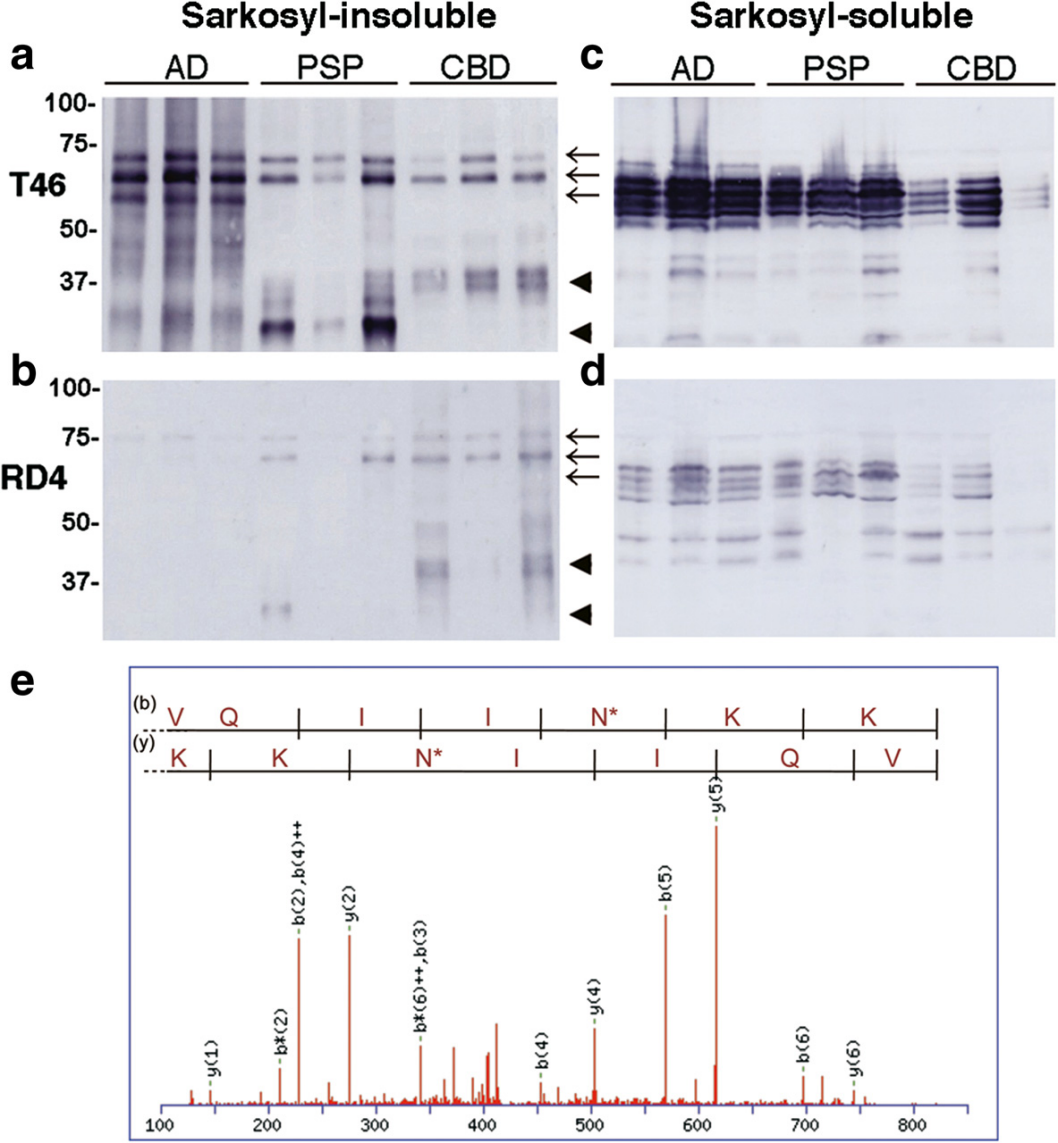
**Immunoblot and LC/MS/MS analyses showed a lower immunoreactivity of RD4 with tau in AD and deamidation at N279.** Immunoblot analysis of Sarkosyl-insoluble **(a, b)** and soluble **(c, d)** tau from AD, PSP and CBD brains (three cases for each disease) with anti-tau monoclonal antibodies T46 **(a, c)** and RD4 **(b, d)**. Arrows indicated the positions of the 60, 64, 68 kDa triplet tau bands in AD brains, and arrowheads indicate the ~33 and ~37 kDa C-terminal fragments that distinguish PSP and CBD. Identification of deamidated amino acid residue by nano-electrospray tandem mass spectrometry **(e)**. Product ion spectrum of a mass signal of tryptic peptide VQIINK detected in Sarkosyl-insoluble tau from AD brain, showing the b and y ion series. These results identify the site of deamidation as N279, indicated by N*.

When Sarkosyl-insoluble fractions of tau from AD, PSP and CBD brains were analyzed by immunoblotting with T46 and RD4, we noticed a lower immunoreactivity of RD4 with abnormal tau in AD compared to that in both PSP and CBD (Figure 1a,b). T46, a monoclonal antibody to the C-terminal region of tau, strongly labeled triplet bands of phosphorylated full-length tau in AD together with smearing substances, and doublet bands together with C-terminal fragments of tau in CBD and PSP (Figure 1a). In contrast, RD4 (1:1000 dilution) stained tau in CBD and PSP relatively strongly, but barely stained the tau bands, and especially the smears, in AD (Figure 1b), though both RD4 and T46 labeled Sarkosyl-soluble tau in these brains (Figure 1c,d). These results suggested that there might be some modification in the RD4 epitope or its vicinity on tau in AD abrogating immunoreactivity. The low affinity of RD4 for tau in AD is consistent with the original report [4], which noted that the RD4 titer appeared to be considerably weaker than those of TP70 and RD3. To confirm that the asparagine residue at position 279 (N279) on tau was deamidated in AD, we performed LC/MS/MS analyses of tryptic peptides of Sarkosyl-insoluble tau prepared from AD brains. As shown in Figure 1e, almost all the VQIINK peptide derived from 4R tau isoform was detected as VQIIN*K (* indicates deamidation) (Figure 1e), while Q276 was normal, strongly suggesting that N279 is extensively deamidated in tau in AD.

### RD4 cannot recognize 4R tau with deamidation at N279

**Figure 2:**
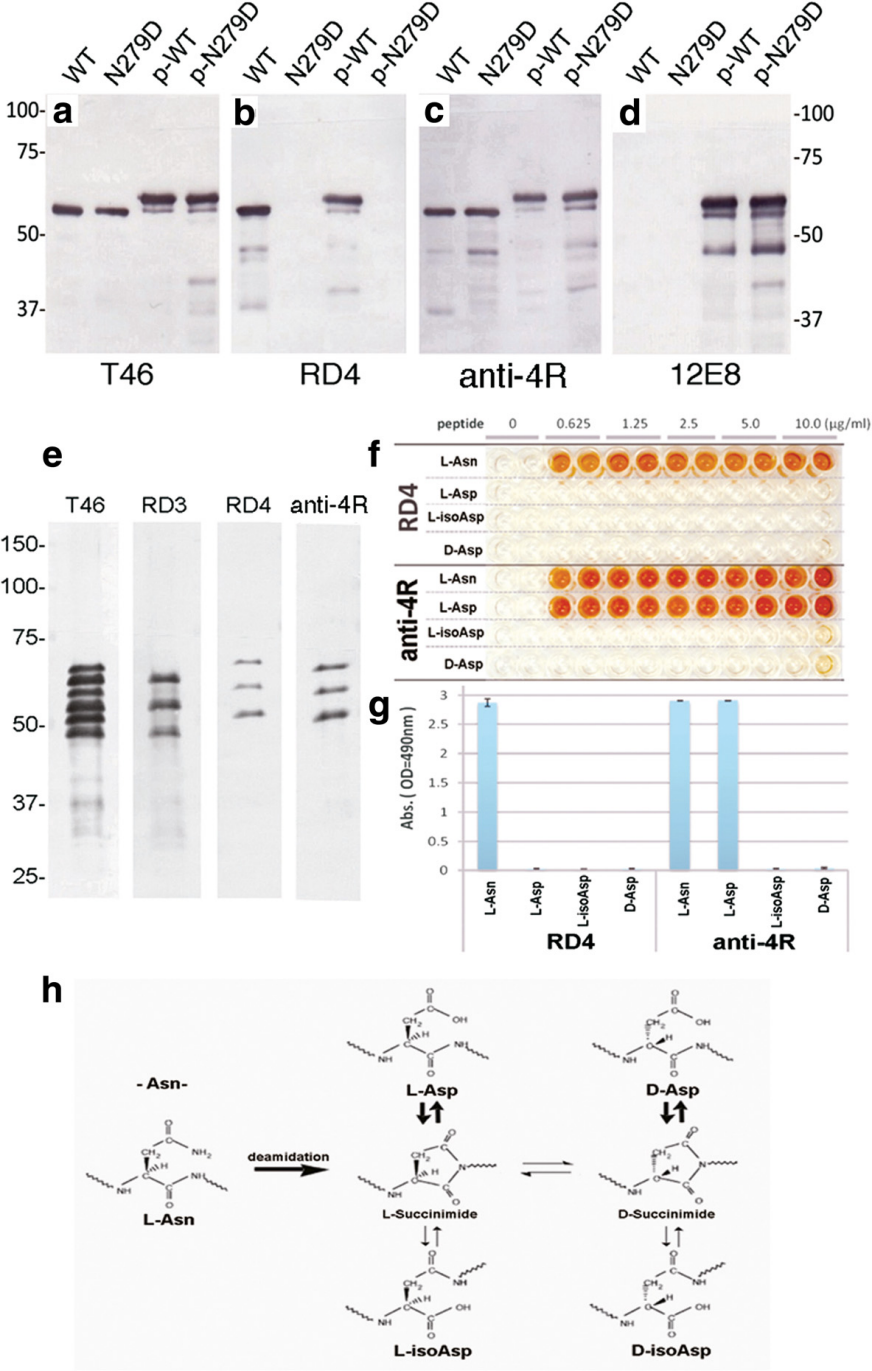
**RD4 cannot recognize N279D-4R tau, but new anti-4R labels both WT and N279D-4R tau equally.** Immunoblot analysis of wild-type (WT) and N279D mutant tau before (WT, N279D) and after (p-WT, p-N279D) phosphorylation with PKA, using T46 **(a)**, RD4 **(b)**, anti-4R **(c)** and 12E8 **(d)** antibodies. Immunoblot analysis of six recombinant human tau isoforms with T46, RD3, RD4 and anti-4R antibodies **(e)**. Specificities of RD4 (1:1000 dilutions) and anti-4R (1:3000 dilutions) antibodies for synthetic peptides, L-Asn (wild-type), L-Asp, L-isoAsp and D-Asp peptides (0.625 ~ 10 μg/mL) tested by ELISA assay **(f)**. Quantitation of the ELISA results (the mean of absorbance at 490 nm on 1.25 μg/mL peptide is shown **(g)**. Pathways for deamidation of asparaginyl residues **(h)**. L-Asn residue can be converted spontaneously via a succinimidyl intermediate to form L-Asp, D-Asp. L-isoAsp and D-isoAsp residue (modified from Ref. [9]).

Since N279 is located in the RD4 epitope, we next examined the effect of deamidation of N279 on immunoreactivity to RD4. Substitution of N279 to aspartic acid was introduced into 4R1N human tau isoform by site-directed mutagenesis, and immunoreactivity to mutant (N279D)-4R tau before and after phosphorylation by protein kinase A was compared with that of wild-type tau. As shown in Figure 2(a-d), RD4 was not able to recognize N279D-4R tau regardless of the phosphorylation state of Ser262, which is located near the RD4 epitope. To further investigate the deamidation of N279 of tau, we immunized a rabbit with a synthetic peptide, VQIIDKKLDLSNVQSKC, which is the RD4 antigen peptide with substitution of N279 to Asp. The antiserum anti-4R labeled both wild-type (WT) and N279D-4R tau equally (Figure 2c) and the immunoreactivity was unaffected by phosphorylation of Ser262/356 with PKA (Figure 2c). The anti-4R antibody specifically bound with recombinant human 4R tau isoforms, as did RD4, but did not react with the 3R tau isoforms (Figure 2e). The specificities of RD4 and anti-4R antibodies were further analyzed by means of ELISA assay using the antigen peptide of RD4 (L-Asn), the peptide with N279D substitution (L-Asp), the peptide with L-isoAsp substitution (L-isoAsp) and the peptide with N279 D-Asp substitution (D-Asp) (Figure 2f,g). These modifications are known to be related to deamidation of Asn residue (Figure 2h). RD4 failed to react with L-Asp (antigen peptide with N279D substitution), whereas anti-4R reacted almost equally with L-Asn (wild-type) and L-Asp peptide. Neither RD4 nor anti-4R reacted with D-Asp or L-isoAsp peptide (Figure 2f,g).

### Antiserum against peptide with deamidation of N279 strongly stained tau smears in AD

**Figure 3:**
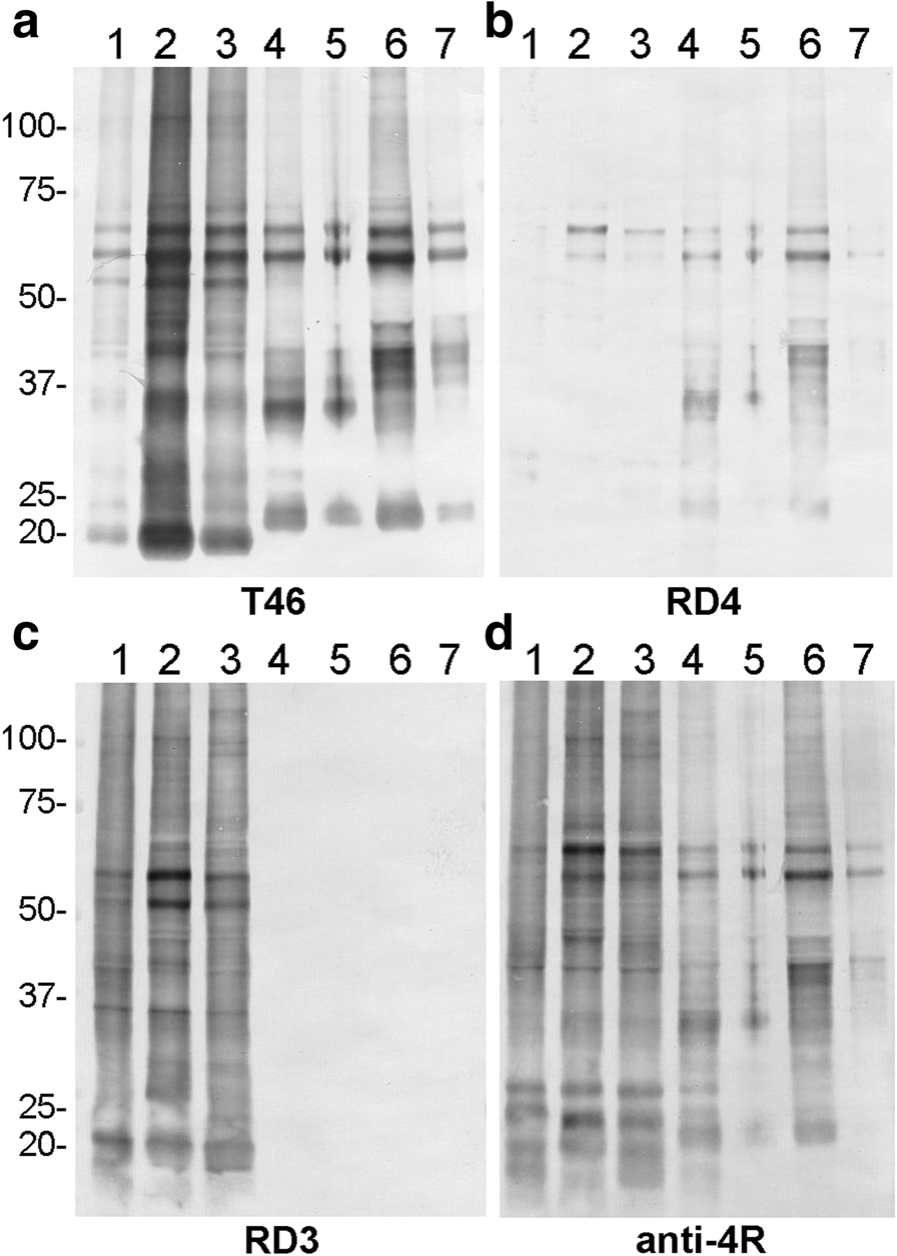
**New anti-4R strongly stained tau bands and smears in AD.** Immunoblot analyses of Sarkosyl-insoluble tau from three AD (1–3), two PSP(4, 5) and two CBD (6, 7) cases with T46 (**a**, 1:2000 dilution), RD4 (**b**, 1:1000 dilution), RD3 (**c**, 1:1000 dilution) and anti-4R antibodies (**d**, 1:2000 dilution).

The immunoreactivity of anti-4R was compared to that of RD4, RD3 and T46 in immunoblotting of Sarkosyl-insoluble tau from tauopathy brains (Figure 3). Samples of insoluble tau from three AD (lane 1–3), two PSP (lane 3, 4) and two CBD (lane 6, 7) cases were examined (Figure 3a-d). RD4 faintly stained only two bands at 64 and 68 kDa in AD brains, whereas it stained several tau fragments in PSP and CBD, in addition to the two bands at 64 and 68 (Figure 3b). In contrast, anti-4R stained tau bands and smears in AD, like RD3, and this staining was much stronger than that of tau bands and smears in PSP and CBD (Figure 3d). These results strongly suggest that tau in AD brains is predominantly deamidated at N279, and that the levels of deamidation are much lower in tau from PSP and CBD brains.

**Figure 4:**
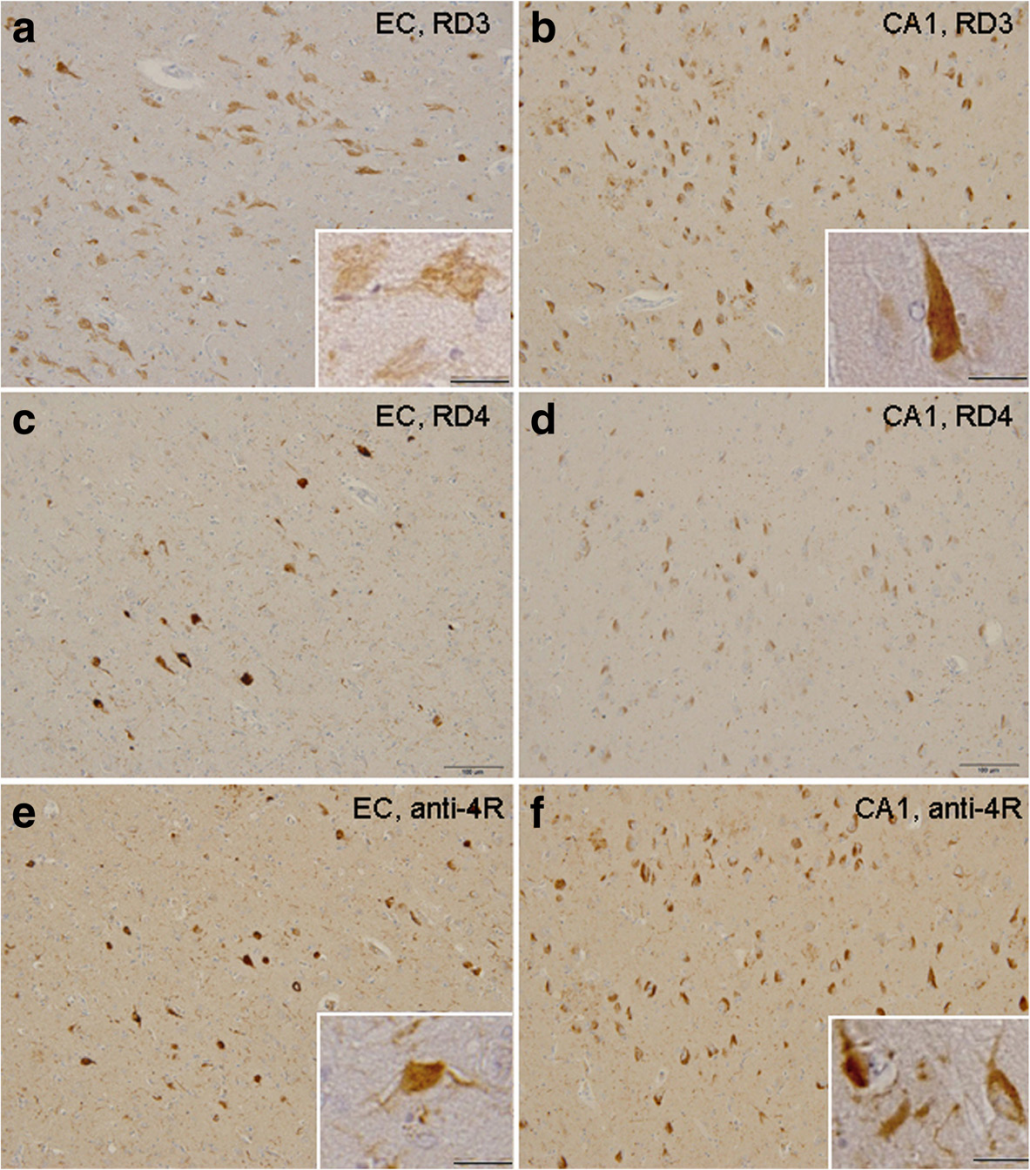
**New anti-4R antibody stained intracellular NFTs more extensively than did RD4.** Immunostaining of entorhinal cortex (EC) and CA1 sections of AD brain after autoclaving and formic acid treatments, using RD3 **(a, b)**, RD4 **(c, d)** and anti-4R **(e, f)** antibodies. NFTs with higher magnification are shown in insets. Bar = 100 μm (25 μm in insets).

Next, we compared the immunostaining of tau pathologies with RD3, RD4 and anti-4R on formalin-fixed brain sections of AD (Figure 4). None of the three antibodies stained tau on formalin-fixed sections in the absence of formic acid treatment or autoclaving (not shown), suggesting that the epitopes of these antibodies are masked. After autoclaving or formic acid treatment, neurofibrillary tangles (NFTs) and neuropil threads (NTs) were detected with these antibodies, and dual treatment with both autoclaving and formic acid strongly enhanced the staining (not shown). The new anti-4R antibody stained intracellular NFTs and NTs more extensively than did RD4 (Figure 4). This was most evident in the CA1 region, where anti-4R strongly stained RD3+/RD4− NFTs (Figure 4d,f). This result indicates that the RD4 epitope is deamidated in pathological tau from AD brain, especially in RD3+/RD4− NFTs. RD3 stained abundant ghost tangles in entorhinal cortex and NFTs in CA1, but failed to stain fine processes of NFTs and NTs (Figure 4), as previously reported [10]. Anti-4R also failed to detect ghost tangles in entorhinal cortex.

### Deamidation of N279 reduces the ability of tau to bind microtubules

**Figure 5:**
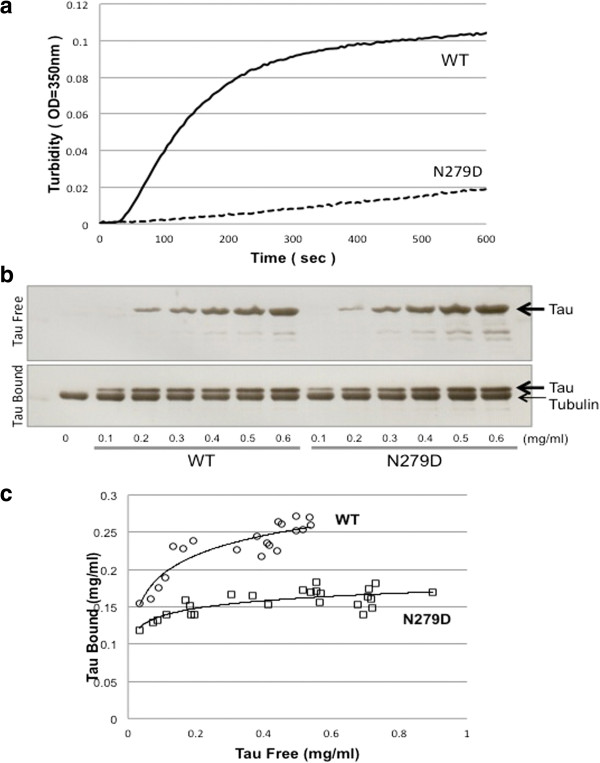
**Deamidation of N279 reduced the functional activity of tau.** Effects of deamidation at N279 on the ability of four repeat htau46 (412 amino acid isoform of human tau) to promote microtubule assembly **(a)** and to bind to microtubules **(b, c)**. **a**, Polymerisation of tubulin induced by wild-type htau46 (solid line) and htau46 N279D (dotted line) was monitored by turbidimetry. Results of a typical experiment are shown; similar results were obtained in three separate experiments. **b**, SDS-PAGE and CBB stainings of free tau and tau bound (arrows) to a constant amount of tubulin (small arrow) are shown. **c**, Scatter plots of quantitations of free tau and tau bound to WT tau (open circles) and N279D mutant tau (open squares).

N279 is located in one of the repeat regions of tau that are involved in binding to microtubules. Therefore, we tested whether the deamidation influences the role of tau in microtubule binding and assembly. First, the ability of N279D mutant tau to mediate polymerization of tubulin was compared to that of wild-type tau by monitoring the turbidity after mixing tubulin with tau. The N279D mutant tau showed a reduced ability to promote microtubule assembly (Figure 5a). We next investigated the binding ability of the N279D mutant tau to taxol-stabilized microtubules. Tau bound and unbound to microtubules was separated by centrifugation and the levels were quantitated by means of SDS-PAGE and CBB staining (Figure 5b,c). When the amount of tau was increased in the presence of a constant amount of microtubules, the binding affinity and the microtubule assembly-promoting activity of N279D mutant tau were both found to be much lower than those of WT tau, clearly indicating that deamidation of N279 reduced the functional activity of tau (Figure 5b,c). Since several positively charged residues have been shown to be important for the ability of tau to promote microtubule assembly, negative charge arising from deamidation of N279 may affect the interaction.

## Discussion

Our present results indicate that the N279 on the RD4 epitope is extensively deamidated in pathological tau from AD brain. Because the widely used RD4 antibody is unreactive to the deamidated epitope, the level of 4R tau isoforms in AD brain will have been markedly underestimated in previous immunohistochemical and biochemical analyses using RD4 antibody. Deamidation is an irreversible, non-enzymatic reaction, in which the amide-containing side chain is removed from asparagine or glutamine. It is known to be a marker for aging in proteins with long life-spans, and, for example, many deamidation sites have been identified in crystallins [11], the major proteins of the eye lens. In biochemical deamidation, the side chain of an asparagine residue attacks the amide group, forming a succinimide intermediate, which, upon hydrolysis, affords either aspartate or isoaspartate [12]. Isoaspartate formation from asparagine residues of tau has been reported in AD brains [9,13], but deamidation has been less well investigated. Nevertheless, deamidation is important because it alters the charge of the amino acid residue, and this can markedly affect protein structure and interaction with other proteins. Therefore, deamidation of N279 may have an effect on tau similar to that of missense mutations in FTDP-tau, many of which affect the ability of tau to promote microtubule assembly or to self-aggregate into amyloid fibrils. Indeed, substitution of N279 to Asp greatly reduced the ability of tau to promote microtubule assembly (Figure 5). However, we did not observe any accelerating effect on tau fibril formation (data not shown). Further studies are needed, but it is possible that the deamidation may be a consequence of aging of tau in paired helical filaments (PHF).

Other potential post-translational modifications in the RD4 epitope include acetylation and methylation on K280 [14-16]. Using antibodies specific for tau acetylated at lysine 280, significant acetylated-tau pathology has been found with a distribution pattern similar to that of hyperphosphorylated tau [15]. However, in our protein chemical analyses (including LC/MS/MS) of AD tau, such modification has not been clearly detected. It is possible that the modification is hardly detectable in LC/MS/MS. But it is also possible that antibodies to acetylated K280 peptide may recognize a tau epitope exposed as a result of conformational change. It remains to be investigated whether the acetylation or methylation alters immunoreactivity to RD4 or whether deamidation of N279 influences immunoreactivity to acetylated K280.

The results of this study have implications for the molecular mechanisms of tau assembly. The RD4 immunoreactivity of AD tau (composed of 3R and 4R tau) is different from that of CBD tau and PSP tau (composed of 4R tau), suggesting that the tau filament core structures may be different. Indeed, abnormal tau filaments characteristic of each disease have been described [17]. It seems reasonable to speculate that the RD4 epitope is integrated in the filament cores in CBD and PSP, making it resistant to deamidation and degradation. However, further analyses will be needed to understand the structures of tau in CBD, PSP and other tauopathies.

Prion-like spreading of intracellular pathological proteins or template (seed)-dependent conversion of normal protein to abnormal forms are candidate molecular mechanisms for involvement in the pathogenesis and progression of neurodegenerative diseases including AD [18-21]. The biochemical and structural differences of tau in AD from that in 4R tauopathies found in this study may therefore have implications for prion-like propagation of tau. Heterodimeric tau composed of both 3R tau and 4R tau with an amyloid-like conformation may act as a template for converting normal 3R and 4R tau to the abnormal structures seen in neurons, forming unique PHF structures composed of both 3R and 4R tau. Therefore, site-specific antibodies are important tools for immunohistochemical and biochemical studies of the role of tau in neurodegenerative diseases.

## Conclusions

We conclude that extensive irreversible post-translational deamidation takes place at asparagine residue 279 **(**N279) in the RD4 epitope of tau in Alzheimer’s disease (AD), but not corticobasal degeneration (CBD) or progressive supranuclear palsy (PSP), and this modification abrogates the immunoreactivity to RD4. An antiserum raised against deamidated RD4 peptide specifically recognized 4R tau isoforms, regardless of deamidation, and strongly stained tau in AD brain.

## Methods

### Human brain tissues

Human brain tissues were obtained from The Manchester Brain Bank, University of Manchester (Manchester, UK), Tokyo Metropolitan Institute of Gerontology (Tokyo, Japan) and NCNP Hospital (Tokyo, Japan). This study was approved by the local research ethics committees of Tokyo Institute of Psychiatry and Tokyo Metropolitan Institute of Medical Science. The subjects included three patients diagnosed with AD, three with PSP and three with CBD, neuropathologically confirmed by immunohistochemistry with antibodies to tau, Aβ, α-synuclein and TDP-43.

### Preparation of sarkosyl-insoluble fractions

Brain samples (0.5 g) from patients with AD, PSP and CBD were each homogenized in 10 ml of homogenization buffer (HB: 10 mM Tris–HCl, pH 7.5 containing 0.8 M NaCl, 1 mM EGTA, 1 mM dithiothreitol). Sarkosyl was added to the lysates (final concentration: 2%), which were then incubated for 30 min at 37°C and centrifuged at 20,000 g for 10 min at 25°C. The supernatant was divided into eight tubes (each 1.3 mL) and centrifuged at 100,000 g for 20 min at 25°C. The pellets were further washed with sterile saline (0.5 mL/tube) and centrifuged at 100,000 g for 20 min. The resulting pellets were used as Sarkosyl-insoluble fraction (ppt).

### LC/MS/MS analysis of sarkosyl-insoluble tau

Sarkosyl-insoluble tau from AD brains was subjected to SDS–PAGE using 4–20% polyacrylamide gel (PAGE mini, Daiichi, Tokyo). After staining with Coomassie brilliant blue R-250 (CBB), the bands corresponding to the phosphorylated tau (64 and 68 kDa) were cut out. In-gel digestion of proteins with 1 μg/ml trypsin was carried out as described previously and the resulting peptides were analyzed by an ion-trap spectrometry (Velos Pro; Thermo Fisher Scientific Inc. Waltham, MA). The MS/MS data files were searched and analyzed using the Mascot Server (Matrix Science Inc., Boston, MA).

### Recombinant tau proteins

Expression constructs for six human tau isoforms in plasmid pRK172 were kindly provided by Dr. Goedert. Site-directed mutagenesis was used to change N279 to Asp (numbering refers to the 441-amino-acid isoform of human brain tau) in the four-repeat 412-amino-acid isoform (expressed from cDNA clone htau46). Wild-type and mutated tau proteins were expressed in *Escherichia coli* BL21(DE3) and purified as described previously[22]. For *in vitro* phosphorylation, purified tau (10 μg/ml) was incubated with PKA (10,000 U/ml; New England Biolabs, Beverly, MA) in 30 mM Tris–HCl buffer (pH 7.5) containing 0.1 mM EGTA, 10 mM MgSO_4_, 0.8 mM PMSF and 2 mM ATP, at 30°C for 1 hr.

### Antibodies

RD3 (directed to residues 209~224: Millipore), RD4 (residues 275~291: Millipore), T46 (residues 404~441: Invitrogen) and pS396 (phospho-Ser396: Calbiochem) were purchased. Antiserum anti-4R was raised against a synthetic peptide VQIIDKKLDLSNVQSKC which corresponds to residues 275~291 of human tau (441 residues), with substitution of N279 to Asp (Sigma Aldrich Japan). The peptide was conjugated to m-maleimidobenzoyl- N-hydrosuccinimide ester-activated keyhole limpet hemocyanin (KLH). The KLH-peptide complex (1 mg of each immunogen) emulsified in Freund’s complete adjuvant was injected subcutaneously into a New Zealand White rabbit, followed by 5 weekly subcutaneous injections of 150 μg KLH-peptide complex emulsified in Freund’s incomplete adjuvant, starting 3 weeks after the first immunization.

### ELISA assay

Each synthetic peptide consisting of residues 275~291 (VQIINKKLDLSNVQSKC) with the fifth position being replaced by L-Asp, L-isoAsp, or D-Asp was synthesized by the solid-phase method (Sigma Aldrich Japan) These peptides, L-Asn (wild-type), L-Asp, L-isoAsp, D-Asp (0.625 ~ 10 μg/ml in 50 mM Tris–HCl, pH 8.8) were coated onto microtitre plates (SUMILON) at 4°C for 16 h. The plates were blocked with 10% fetal bovine serum (FBS) in PBS, incubated with the first antibodies (RD4, 1:1000; anti-4R, 1:3000) diluted in 10% FBS/PBS at room temperature for 1.5 h, followed by incubation with HRP-goat anti-rabbit IgG (Bio-Rad) at 1:1000 dilution, and reacted with the substrate, 0.4 mg/ml o-phenylendiamine, in citrate buffer (24 mM citric acid, 51 mM Na_2_HPO_4_), The absorbance at 490 nm was measured using Plate Chameleon (HIDEX) as described [23].

### Microtubule assembly and tau binding

Purified recombinant wild-type and mutant tau (htau46) proteins (0.1 mg/ml, 2.3 μM) were incubated with bovine brain tubulin (1 mg/ml, 20 μM, cytoskeleton) in assembly buffer at 37°C, as described [22]. The assembly of tubulin was monitored in terms of the change in turbidity at 350 nm. The binding assay was performed as described [24]. Briefly, purified tubulin was incubated at 37°C in the presence of 1 mM GTP and 20 μM taxol. Tau protein was added at various concentrations and each mixture was incubated for 10 min. The suspensions were centrifuged for 100,000 g at 37°C. The resulting pellets were resuspended in 50 mM PIPES pH 6.9, 1 mM EGTA, 0.2 mM MgCl_2_, 5 mM DTT, 0.5 M NaCl. The pellets and supernatants (containing bound and free tau, respectively) were subjected to SDS-PAGE and stained with Coomassie brilliant blue R250. The gels were scanned at 400 dpi on a gel scanner and evaluated using the software provided.

### Gel electrophoresis and immunoblotting

Samples were run on gradient 4-20% or 10% polyacrylamide gels and electrophoretically transferred to PVDF membranes. Residual protein-binding sites were blocked by incubation with 3% gelatin (Wako) for 10 min at 37°C, followed by overnight incubation at room temperature with the primary antibody. The membrane was then incubated for 1 hr at room temperature with anti-rabbit IgG (BA-1000, Vector Lab) or anti-mouse IgG (BA-2000, Vector lab), then incubated for 30 min with avidin-horseradish peroxidase (Vector Lab), and the reaction product was visualized by using 0.1% 3,3-diaminobenzidine (DAB) and 0.2 mg/ml NiCl_2_ as the chromogen.

### Immunohistochemistry

Formalin-fixed paraffin-embedded sections of AD brains were used for immunohistochemistry. The sections were pretreated by autoclaving for 10 min in 10 mM sodium citrate buffer at 120°C and treated with 100% formic acid for 10 min. Sections were washed with 10 mM phosphate-buffered saline (PBS, pH 7.4) three times for 10 min each. Sections were blocked with 10% normal serum and incubated overnight at room temperature with one of the primary antibodies in PBS. After washing, sections were incubated with biotinylated anti-mouse or rabbit secondary antibody for 2 h, followed by biotinylated horseradish peroxidase complex (ABC, Vector) for 1 hr. The label was visualized with EnVision™(Dako). Sections were counterstained with hematoxylin.

## Competing interests

The authors declare that they have no competing interests.

## Authors’ contributions

AD, MT, MS and TN performed biochemical and immunochemical studies. FK performed LC/MS/MS analysis. HK performed immunohistochemistry. TA helped for characterisation of antibody. HA, YS, HH, SM and DM performed neuropathological studies and analyses. MH performed study design, preparation of antibody, biochemical analyses and wrote the paper. All authors read and approved the final manuscript.
